# Composites Based on Gellan Gum, Alginate and Nisin-Enriched Lipid Nanoparticles for the Treatment of Infected Wounds

**DOI:** 10.3390/ijms23010321

**Published:** 2021-12-28

**Authors:** Katarzyna Reczyńska-Kolman, Kinga Hartman, Konrad Kwiecień, Monika Brzychczy-Włoch, Elżbieta Pamuła

**Affiliations:** 1 Department of Biomaterials and Composites, Faculty of Materials Science and Ceramics, AGH University of Science and Technology, Al. Mickiewicza 30, 30-059 Kraków, Poland; kkwiecien@agh.edu.pl; 2 Department of Analytical Chemistry and Biochemistry, Faculty of Materials Science and Ceramics, AGH University of Science and Technology, Al. Mickiewicza 30, 30-059 Kraków, Poland; kinga.piechura24@gmail.com; 3 Department of Molecular Medical Microbiology, Faculty of Medicine, Medical College, Jagiellonian University, Ul. Czysta 18, 31-121 Kraków, Poland; m.brzychczy-wloch@uj.edu.pl

**Keywords:** solid lipid nanoparticles, nisin, antibacterial wound dressings

## Abstract

Due to growing antimicrobial resistance to antibiotics, novel methods of treatment of infected wounds are being searched for. The aim of this research was to develop a composite wound dressing based on natural polysaccharides, i.e., gellan gum (GG) and a mixture of GG and alginate (GG/Alg), containing lipid nanoparticles loaded with antibacterial peptide—nisin (NSN). NSN-loaded stearic acid-based nanoparticles (NP_NSN) were spherical with an average particle size of around 300 nm and were cytocompatible with L929 fibroblasts for up to 500 µg/mL. GG and GG/Alg sponges containing either free NSN (GG + NSN and GG/Alg + NSN) or NP_NSN (GG + NP_NSN and GG/Alg + NP_NSN) were highly porous with a high swelling capacity (swelling ratio above 2000%). Encapsulation of NSN within lipid nanoparticles significantly slowed down NSN release from GG-based samples for up to 24 h (as compared to GG + NSN). The most effective antimicrobial activity against Gram-positive *Streptococcus pyogenes* was observed for GG + NP_NSN, while in GG/Alg it was decreased by interactions between NSN and Alg, leading to NSN retention within the hydrogel matrix. All materials, except GG/Alg + NP_NSN, were cytocompatible with L929 fibroblasts and did not cause an observable delay in wound healing. We believe that the developed materials are promising for wound healing application and the treatment of bacterial infections in wounds.

## 1. Introduction

Wound healing is a complicated, multi-step process involving inflammation, proliferation, migration, and remodeling of new tissue [1,2]. The presence of pathogenic bacteria at the wound site and subsequent development of bacterial infection are recognized as major factors compromising this process [3]. Bacteria can delay the healing procedure at best but can even lead to systemic infection and death at worst [1,4]. Opportunistic bacteria, such as *Staphylococcus aureus*, *Streptococcus pyogenes*, *Escherichia coli*, or *Pseudomonas aeruginosa*, easily colonize the wound site and create a protective biofilm, which makes the infection even more challenging to cure [5,6,7].

Topical application of antimicrobials, e.g., in the form of a controlled drug release dressing, is commonly utilized to eradicate bacteria and facilitate wound healing [3,8,9]. Natural polysaccharides are one of the most commonly used materials for the fabrication of novel wound dressings due to their excellent biocompatibility, high swelling capacity, low cost, and presence of numerous functional groups available for prospective modifications [10]. Among them, alginate (Alg) gained increased interest as it is fully biocompatible, can be processed into different forms (e.g., electrospun fibers, porous sponges, or films) and, as it is usually cross-linked with Ca^2+^, it can exchange ions with body fluids (namely Na^+^) and thus contribute to hemostasis [10]. Alg has been already used for the production of gentamicin, vancomycin, or minocycline-loaded hydrogels. It was evident that upon application, the antibiotics were released from hydrogels efficiently over time, quickly penetrated the wound, prevented the spread of bacterial infection, and facilitated wound healing in the porcine burn wound model [11]. A mixture of Alg and chitosan was also used for the production of electrospun nanofiber dressings containing gentamicin. As proved by Bakhsheshi-Rad et al. [12], obtained materials were able to release antibiotic cargo for up to ten days, have an antibacterial effect against *S. aureus* and *E. coli*, and significantly reduce wound healing time in the Balb/C mice model. Mahmood et al. [13] utilized gellan gum (GG), a polysaccharide produced by an aerobic fermentation by *Pseudomonas elodea*, for the fabrication of ofloxacin and lavender-oil-loaded hydrogel films for wound dressing applications. In vivo tests performed using an excision wound model in rats proved that the developed materials facilitated wound closure due to reduction in inflammation, bacteria eradication, and maintaining a proper moist environment. Zhang et al. [14] prepared double cross-linked composite wound dressings based on carboxymethyl chitosan and GG microspheres with a sufficient swelling ratio and stability. The inclusion of antibiotics (i.e., tetracycline hydrochloride and silver sulfadiazine) in GG microspheres provided their sustained release, and in consequence, the materials displayed an excellent antibacterial activity against *E. coli* and *S. aureus*.

However, bacteria with very short generation times (even about 20 min) and a wide variety between species can develop resistance to antibiotics in little time [15]. As antibiotics are in general overused or misused, many multi-drug resistant bacteria have already been identified [16,17]. It seems like antibiotics are being overpowered by the bacteria and their ability to evolve. Although several antibiotic-releasing wound dressings have been developed, their efficacy might diminish, especially in patients with chronic wounds colonized by antibiotic-resistant pathogens. Thus, novel antimicrobial strategies are needed in the field of wound healing.

Our attention was drawn to antibacterial peptides (ABPs) as a promising alternative for antibiotics. ABPs are cationic macromolecules consisting of 12–45 amino acids. Depending on their composition, ABPs are effective against a broad range of bacterial species (both Gram-negative and Gram-positive), fungi, and even viruses. They are also able to destroy multi-drug-resistant bacteria that cannot be eradicated by antibiotics. The bacterial resistance for ABPs can be induced only in very specific conditions (including multiple exposures to sub-inhibitory concentrations of ABPs) [18]. The first known ABP was nisin (NSN) isolated from *Lactococcus lactis* in 1947. It has 34 amino acids and is highly active against Gram-positive bacteria (including *Listeria monocytogenes*, *Bacillus cereus*, and multidrug-resistant strains of *Staphylococcus aureus*). Positively charged NSN binds to negatively charged bacteria inducing the formation of pores in the cell membrane and leakage of ATP, amino acids, and loss of ion gradients, which in consequence leads to bacterial death [19,20]. Mouritzen et al. [21] proved that NSN could decrease bacterial growth and facilitate wound healing by decreasing the concentration of proinflammatory cytokines (i.e., interleukin-6 and interleukin-8). It also reduced the levels of lipopolysaccharide-induced tumor necrosis factor-α. NSN was already used by Gunes et al. [22] for the production of carboxymethyl chitosan/alginate-impregnated cotton wound dressings. Fabricated materials had a high fluid absorption capacity, good mechanical properties, and showed antibacterial activity against *S. aureus*.

Although ABPs are regarded as a new generation of antibiotics and a good alternative for them, several issues must be solved before ABPs can be used in clinics [23]. To enhance the efficacy of ABPs, protect them from premature degradation or inactivation, and provide control over their release, they can be encapsulated in a carrier (e.g., solid lipid nanoparticles) and delivered directly to the infected site. Solid lipid nanoparticles can be made of various types of biocompatible lipids, including fatty acids, glycerol esters, waxes, or sterols [24]. Depending on the lipid used, it is possible to effectively encapsulate both lipophilic and hydrophilic drugs [24,25]. Lipid nanoparticles can be used in the treatment of infected wounds. Topical formulations based on lipid nanoparticles were proved to increase the stability of various drugs and to increase penetration of active pharmaceutical ingredients through the skin (in comparison to the free drug) [26].

Ghaffari et al. [27] fabricated solid lipid nanoparticles loaded with curcumin and ampicillin using high-pressure homogenization methods. The nanoparticles, further used in the form of ointment or gel formulation, demonstrated antibacterial efficacy against *S. aureus* and *P. aeruginosa* and significantly increased the wound healing rate in the rat skin burn model. Lipid nanoparticles can also be incorporated into hydrogels or electrospun dressings, as evidenced by Sand et al. [28]. The developed chitosan–hyaluronic acid sponge dressings containing lipid nanoparticles loaded with andrographolide were highly porous, with proper swelling capacity, and were able to release drug cargo for up to 72 h.

Alg or GG are regarded as promising materials for the fabrication of spongy wound dressings, but unfortunately, on their own, they do not possess any antimicrobial activity. We hypothesize that the inclusion of nisin in a wound dressing based on Alg and GG will provide those materials with antibacterial properties. However, as preparation of those hydrogels requires relatively high temperatures [29], direct addition of thermally labile NSN to heated solution might lead to polypeptide denaturation, and in consequence, loss of its antibacterial efficacy. Thus, this study aimed to: (1) purify commercially available nisin salt to obtain free NSN with increased NSN content and retained antibacterial activity, (2) encapsulate purified NSN in lipid nanoparticles to protect from thermal denaturation of NSN, and (3) use the developed nanoparticles as antibacterial agents in composite hydrogel-based wound dressing. The novelty of the proposed solution is in: (1) the use of antibacterial peptide—NSN, which is less prone to bacterial resistance than the conventional antibiotics, and (2) inclusion of NSN-loaded nanoparticles in hydrogel dressing for thermal protection and controlled release of the peptide.

## 2. Results

The following study focused on the fabrication of composite spongy wound dressings based on hydrogels (i.e., GG and a mixture of GG/Alg) containing lipid nanoparticles loaded with antibacterial peptide—nisin (NSN).

**Figure 1:**
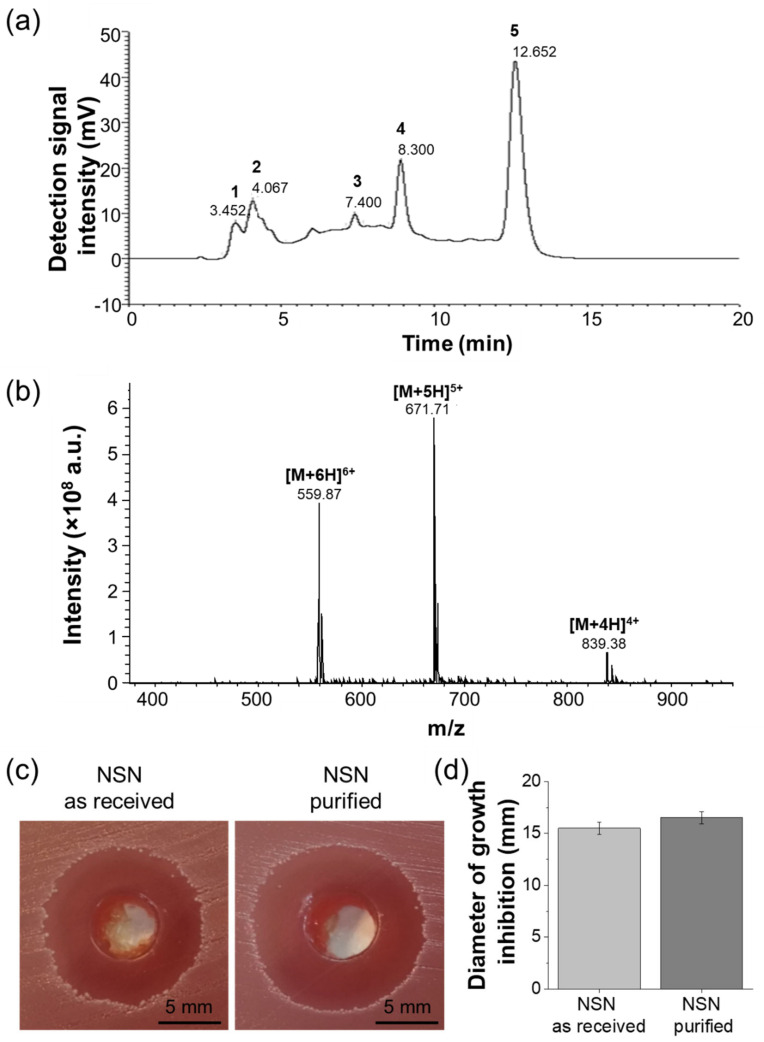
Nisin purification: HPLC chromatogram of NSN after reverse-phase purification (**a**), the mass spectrum of NSN (**b**), pictures of *S. pyogenes* growth inhibition zones (**c**) and diameters of *S. pyogenes* growth inhibition zones (**d**).

Prior to nanoparticle manufacturing, commercially available NSN was purified using HPLC. This process was highly effective and allowed us to obtain high-quality NSN. NSN, after reverse-phase purification, exhibited absorbance at 215 nm (Figure 1a). Eluted fractions were collected and characterized using ESI-MS. According to the obtained results, NSN was identified in fraction 5. The mass spectrum showed the presence of multiple charged ions of expected mass: [M + 4H]^4+^ = 839.38, [M + 5H]^5+^ = 671.71 and [M + 6H]^6+^ = 559.87, which are specific to nisin (Figure 1b).

The preliminary studies on the antibacterial efficacy of NSN were performed using *S. pyogenes*. The bacterial growth inhibition zones formed around the wells containing NSN solutions were observed for both as-received NSN and purified NSN (Figure 1c). The diameters of growth inhibition zones were 15.5 ± 0.6 mm for NSN as-received (before purification) and 16.5 ± 0.6 mm for purified NSN (Figure 1d). The observed difference was not statistically significant. Thus, the purified NSN was used in all further experiments.

**Figure 2:**
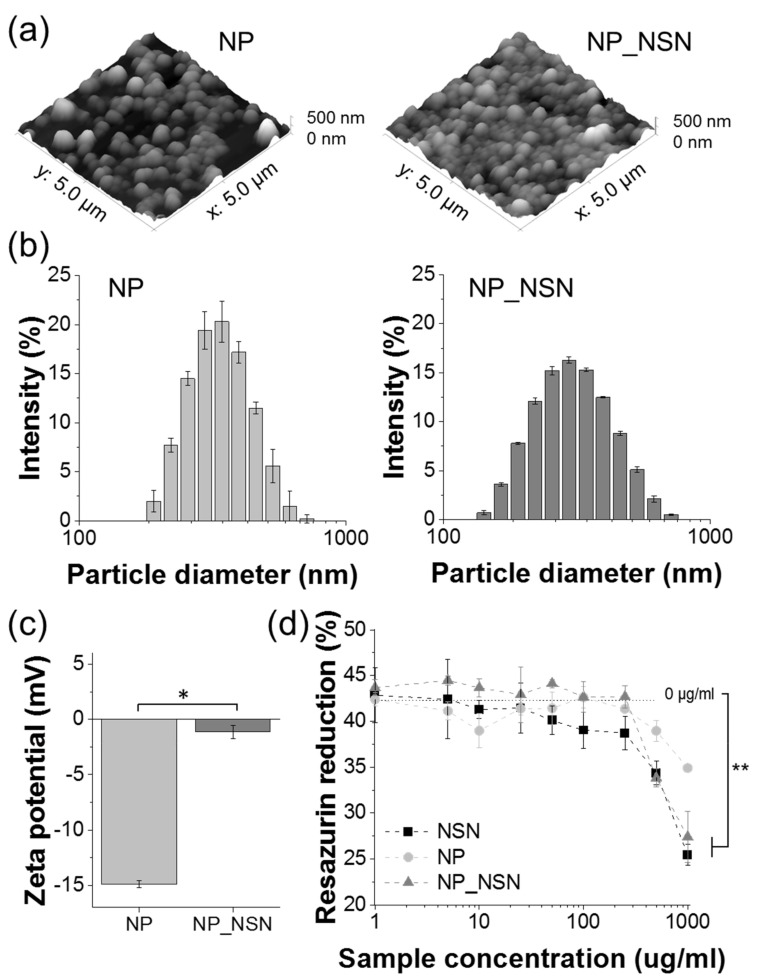
Characterization of unloaded NP and NSN-loaded NP: AFM images (**a**), particle size distribution (**b**), surface zeta potential (**c**), and metabolic activity of L929 fibroblasts incubated for 24 h in presence of unloaded and NSN-loaded NP (**d**). Statistically significant differences at * *p* < 0.05 and ** *p* < 0.01.

Stearic acid-based solid lipid nanoparticles manufactured using the double emulsification/solvent evaporation method were spherical as evaluated by AFM (Figure 2a). The presence of NSN did not influence particle shape, neither no irregular particles nor NSN crystals were observed. The average particle size determined by DLS (Figure 2b) was 322.5 ± 3.7 nm for NP and 297.3 ± 4.2 nm for NP_NSN. Thus, the presence of NSN in NP caused a slight decrease in particle size. It also slightly increased polydispersity index (PdI), as unloaded NP were highly uniform with PdI of 0.088 ± 0.028, while PdI for NP_NSN was equal to 0.152 ± 0.023. No particles or particle agglomerates exceeding 750 nm were found in both types of NP. Surface zeta potential (Figure 2c) changed significantly upon encapsulation of NSN. Unloaded NP were characterized by strongly negative surface charges (−14.9 ± 0.3 mV), while the addition of positively charged NSN increased the surface zeta potential of the NP_NSN to −1.1 ± 0.6 mV.

According to the OPA-based amine detection method, NSN encapsulation efficacy in NP_NSN was equal to 81.9 ± 4.3%, resulting in NSN loading of 5.2 ± 0.3% (meaning that on average 52 µg of NSN was present in 1 mg of NP_NSN).

The developed NP and NP_NSN, along with free NSN, were tested in contact with L929 fibroblasts to determine their cytotoxicity at a wide range of concentrations (i.e., 1–1000 µg/mL) (Figure 2d). Unloaded NP did not exhibit any significant cytotoxicity, although the highest NP concentration (1000 µg/mL) caused a 17% decrease in cell viability as compared to untreated cells (0 µg/mL). NSN alone and NP_NSN were cytotoxic for L929 cells at 1000 µg/mL concentration (60% and 65% of the viability of untreated cells, respectively). Lower concentrations did not influence cell viability.

NSN-loaded NP (NP_NSN) were further used for the fabrication of composite hydrogel-based wound dressings. GG and a mixture of GG and Alg at a 1:1 mass ratio (GG/Alg) were used as composite matrix, while NSN or NP_NSN were added to append antibacterial properties. The amount of NSN, either alone or encapsulated in NSN_NP, was equal in all samples. All samples underwent gelation and retained their shape after cutting into samples of desired sizes and shapes.

**Figure 3:**
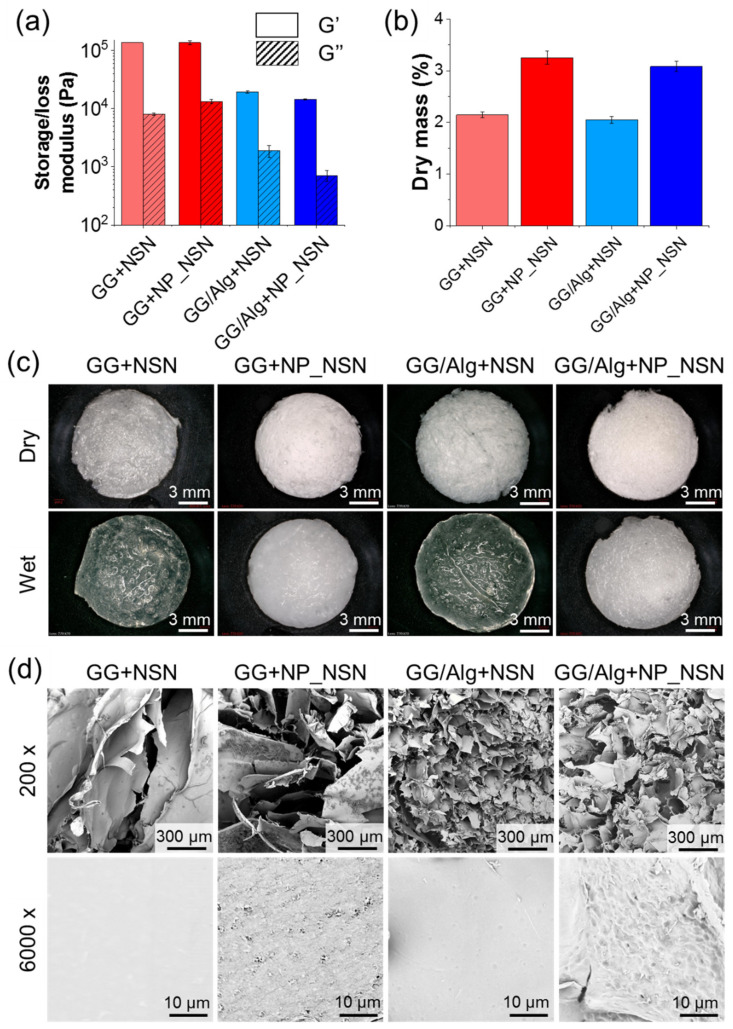
Physico-chemical characterization of GG and GG/Alg hydrogels containing NSN or NP_NSN: rheological characteristics (**a**), dry mass percentage (**b**), gross morphology in dry and hydrated (wet) state (**c**) and SEM images at magnification of 200× and 6000× (**d**).

The rheological properties of the developed composites (Figure 3a) differed significantly between GG and GG/Alg. The average storage moduli (G′) for GG + NSN or GG + NP_NSN were 1.38 × 10^5^ Pa and 1.37 × 10^5^ Pa, respectively. The presence of NSN_NP increased the loss modulus (G″) to 1.3 × 10^4^ Pa for GG + NP_NSN, as compared to 8.1 × 10^3^ Pa for GG_NSN. In the case of GG/Alg-based samples, both G′ and G″ were significantly decreased in comparison to the analog GG samples (around a 10-fold reduction).

No significant differences in G′ were observed between GG/Alg + NSN and GG/Alg + NP_NSN (2.0 × 10^4^ Pa and 1.5 × 10^4^ Pa, respectively). However, the addition of NP_NSN decreased G″ in GG/Alg + NP_NSN (7.1 × 10^2^ Pa) in comparison to GG/Alg + NSN (1.9 × 10^3^ Pa).

Dry mass content (Figure 3b) in NSN-containing samples was around 2.14 ± 0.06% for GG + NSN and 2.05 ± 0.06% for GG/Alg + NSN. It corresponds well to calculated theoretical solid content in those samples, i.e., 2% *w*/*v* originating from hydrogel, 0.1% *w*/*v* coming from CaCl_2_ and 0.052% *w*/*v* from NSN addition. The presence of 1% *w*/*v* NP_NSN increased dry mass content in GG + NP_NSN to 3.25 ± 0.13% and to 3.08 ± 0.10% in GG/Alg + NP_NSN.

Macroscopically, freeze-dried samples (Figure 3c, top panel) were characterized by a spongy-like appearance and could be easily cut into the desired form. The presence of NP_NSN provided GG + NP_NSN and GG/Alg + NP_NSN with some whiter coloring. Upon hydration for 1 h in PBS (Figure 3c, bottom panel), the samples retained their shape, no visible dissolution or damage to sample integrity was observed. NSN-containing samples became more translucent while NP_NSN-loaded hydrogels remained white and opaque.

SEM analyses (Figure 3d, top panel) revealed that the inner microstructure of all samples was highly porous. However, the pores observed in GG-based samples were much larger (100–500 µm) and less uniform than in the case of GG/Alg materials (below 100 µm). Although not many NP_NSN or nanoparticle agglomerates were visible within the pores, it was observed that at higher magnification (Figure 3d, bottom panel) the pore surfaces of NP_NSN-loaded hydrogels were coarser, with distinct irregularities, in comparison to the smooth surface of the pore surfaces present in NSN-loaded samples. The observed roughness was attributed to the presence of NP_NSN within the structure of freeze-dried hydrogels.

**Figure 4:**
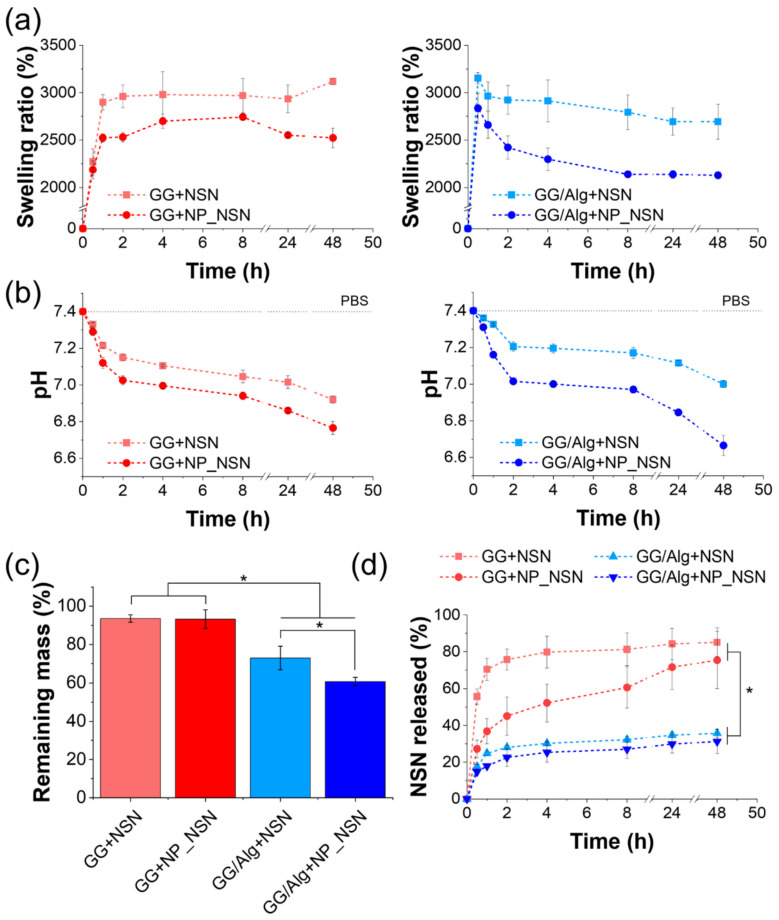
Swelling ratio (**a**) and pH change (**b**) during the incubation in PBS buffer, mass loss after 48 h of incubation (**c**) and NSN release profile (**d**) of GG and GG/Alg hydrogels containing NSN or NP_NSN. Statistically significant differences at * *p* < 0.05.

As the fabricated wound dressings were designed for the treatment of infected wounds in which the exudate is commonly present, their behavior in the water environment was also evaluated. The swelling capacity of the samples was evaluated via immersion in PBS at 37 °C for up to 48 h (Figure 4a). It was evidenced that GG/Alg-based materials were able to absorb more buffer within the first 30 min of incubation than GG alone. Nonetheless, after 1 h, all the samples were uptaken between 2520 ± 40% (GG + NP_NSN) and 2960 ± 150% (GG/Alg + NSN) of PBS in comparison to their initial mass. The swelling capacity of GG + NSN and GG + NP_NSN remained at roughly the same level throughout the whole experiment, while a significant decrease in swelling was observed for GG/Alg + NSN and GG/Alg + NP_NSN. The difference was more pronounced for GG/Alg + NP_NSN (swelling after 48 h was 2694 ± 184% for GG/Alg + NSN and 2128 ± 11% for GG/Alg + NP_NSN).

The pH value of PBS used for sample incubation decreased in the course of time (Figure 4b). The most distinct drop was observed in all samples within the initial 2 h (7.15 ± 0.02 for GG + NSN, 7.03 ± 0.02 for GG + NP_NSN, 7.21 ± 0.03 for GG/Alg + NSN, and 7.02 ± 0.02 for GG/Alg + NP_NSN). In the case of GG/Alg + NP_NSN, further reduction was observed for up to 48 h (final pH equal to 6.67 ± 0.05). Regardless of hydrogel type, the more pronounced differences between the initial pH and pH during incubation were observed for NP_NSN-containing samples.

After 48 h of incubation in PBS for swelling and pH measurements, the samples were weighted for evaluation of the remaining mass (Figure 4c). The slowest degradation was registered for GG-based materials. The remaining mass of those samples (i.e., 93.5 ± 2.0% for GG + NSN and 93.3 ± 4.8% for GG + NP_NSN) was significantly higher than in GG/Alg-based samples. The incubation caused 27% mass loss in GG/Alg + NSN (remaining mass of 73.0 ± 6.1%) and an even more pronounced 40% loss in GG/Alg + NP_NSN (remaining mass of 60.7 ± 2.2%).

NSN release from the developed composite dressings was evaluated using an OPA-based amine detection assay (Figure 4d). Significant differences in NSN release profiles were observed between GG and GG/Alg. In the case of GG + NSN, 55.7 ± 4.2% of NSN was released from the system in the first 30 min of incubation, while more than 80% of NSN was released within 8 h (81.2 ± 8.9%). Further incubation resulted in the release of low NSN doses (85. 1 ± 7.8% NSN released for up to 48 h). Encapsulation of NSN within lipid nanoparticles slowed down initial NSN release from GG + NP_NSN as only 27.3 ± 4.8% of NSN was released in the first 30 min, followed by the gradual and uniform release of NSN for up to 24 h (71.6 ± 12.0%). Subsequent incubation for the next 24 h led to the release of less than 4% of NSN (in total 75.4 ± 15.4% of NSN was released in 48 h). Surprisingly, the release of NSN from GG/Alg matrix was noticeably lower, as only 35.7 ± 2.3% and 31.2 ± 6.6% of NSN was released from the GG/Alg + NSN and GG/Alg + NP_NSN, respectively. No significant differences in NSN release profiles were observed between GG/Alg + NSN and GG/Alg + NP_NSN.

**Figure 5:**
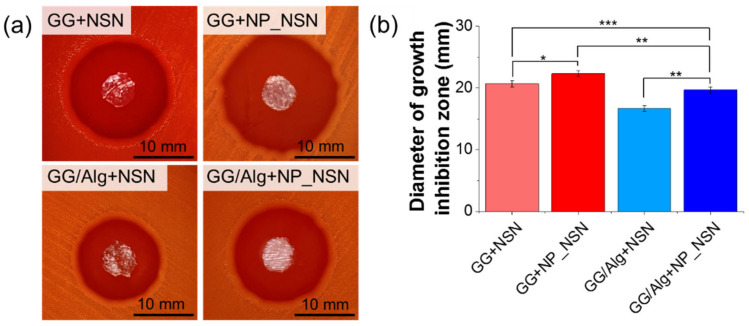
Antibacterial efficacy of GG and GG/Alg hydrogels containing NSN or NP + NSN: pictures of *S. pyogenes* growth inhibition zones (**a**) and diameters of *S. pyogenes* growth inhibition zones (**b**). Statistically significant differences at * *p* < 0.05, ** *p* < 0.01 and *** *p* < 0.001.

The antibacterial efficacy of the composite wound dressings was tested in contact with *S. pyogenes*. In all cases, significant bacteria growth inhibition zones were visible (Figure 5a). NSN release and diffusion in agar was possible since freeze-dried samples had uptaken water from agar substrate. Hydrated samples of NSN-loaded hydrogels became more translucent in comparison to NP_NSN-containing ones. The average diameter of the growth inhibition zone was 20.7 ± 0.5 mm for GG + NSN, while for GG + NP_NSN it was 22.3 ± 0.5 mm (Figure 5b). Incubation of *S. pyogenes* with GG/Alg-based samples resulted in lower inhibition zones, i.e., 16.7 ± 0.5 mm for GG/Alg + NSN and 19.7 ± 0.5 mm for GG/Alg + NP_NSN. The growth of *S. pyogenes* was not influenced by the presence of PBS (negative control, data not shown).

**Figure 6:**
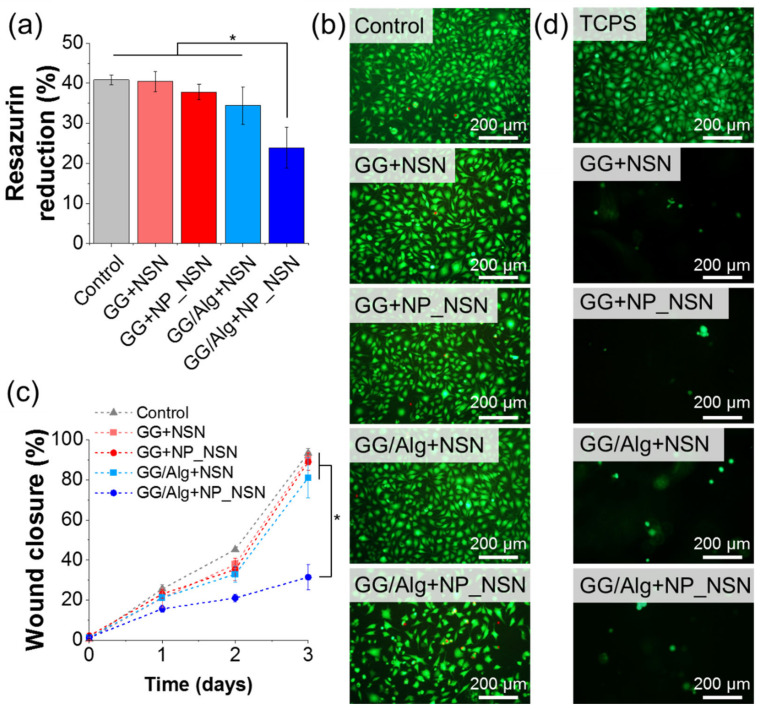
Cytocompatibility of GG and GG/Alg hydrogels containing NSN or NP + NSN: metabolic activity (**a**) and live/dead staining (**b**) of L929 cells incubated for 24 h in presence of sample extracts, wound healing assay performed for up to 3 days in presence of sample extracts (**c**) and evaluation of cell adhesion to the samples 24 h after cell seeding (**d**). Statistically significant differences at * *p* < 0.05.

The final evaluation of the developed composite wound dressings focused on their cytocompatibility with L929 fibroblasts. Metabolic activity of cells determined using resazurin reduction assay (Figure 6a) was not significantly influenced by the presence of sample extracts (84–99% viability of control, i.e., untreated cells), except from GG/Alg + NP_NSN (56% viability of control). Those measurements were confirmed by live/dead staining (Figure 6). The cells treated with GG + NSN, GG + NP_NSN, and GG/Alg + NSN extracts did not differ in morphology from control cells. Only single dead cells stained in red were visible (less than 2%). However, in the case of GG/Alg + NP_NSN, the number of cells was noticeably lower in comparison to control. Although the majority of the cells remained viable, some of them were found to be more rounded, which may indicate an early apoptotic state.

A wound closure assay (Figure 6c) was performed to estimate how the presence of the composite wound dressing would influence the healing of the infected wound. The initial area of a scratch was covered by cells in less than 2.1% (caused by the presence of the cells that were not detached from the surface or not removed from the scratch during the rinsing procedure with PBS). Upon 24 h incubation, the cells started to migrate towards the center of the scratch and covered between 21.1 ± 4.8% (GG + NSN) to 25.6 ± 2.3% (control) of the wound, except for GG/Alg + NP_NSN in which case the wound was closed in only 15.6 ± 1.7%. Over time, the cells scarred over the scratch, and by day three of post-treatment, the wounds were closed by more than 80%. Maximal wound closure measured for GG/Alg + NP_NSN after three days was equal to 31.4 ± 6.2%, which was found to be significantly lower in comparison to control and other samples.

Cell adhesion assay (Figure 6d) showed a scarce tendency of the cells for adhesion to material surface after 24 h post-seeding. Control cells were seeded on TCPS and those cells adhere perfectly to the substrate, while only single cells were found on the surface of all composite wound dressings. Nonetheless, the observed cells were viable, which was evidenced by green staining with calcein AM, no dead cells stained in red by propidium iodide were visible.

## 3. Discussion

As pathogenic bacteria relatively quickly gain resistance to antibiotics, novel antimicrobial strategies are needed for the treatment of bacterial infection in wounds. Thus, our study was aimed at the development of composite hydrogel-based wound dressing loaded with lipid NP containing antibacterial peptide—NSN. GG and a mixture of GG/Alg were selected for the fabrication of spongy wound dressing since they are well known for their biocompatibility, fluid absorption capacity, and ease of processing. Lipid NP provided sustained release of NSN and protection against thermal deactivation of NSN during mixing with heated solutions of GG and GG/Alg during sample preparation.

Commercially available NSN formulation contains only 2.5% *w*/*w* active pharmaceutical ingredient (NSN), which is highly unfavorable in terms of its encapsulation within any drug carrier. Less substrate to be encapsulated in NP should result in more efficient drug loading and enhance carrier activity. To increase encapsulation efficacy and further concentration of an active ingredient within NP, NSN was purified using HPLC. Fractions present in commercially available NSN powder were separated, and based on the research performed by Holcapkova et al. [30], NSN was identified in fraction 5. Previous studies by Taylor et al. [31] proved that extraction in organic solvent (i.e., methanol or ethanol) might also increase NSN concentration. However, the developed method was way less effective than HPLC as it was possible to recover up to 75% of NSN with the proper combination of solvents. Apart from a significant increase in NSN concentration, the procedure also removed any unnecessary components present in the commercial reagent (e.g., excess NaCl), which contributes to the increased quality and safety of NSN. However, since NSN is a polypeptide, the major concern related to its purification process was connected to its stability and antibacterial efficacy. Thus, before the application of purified NSN in the production of NP_NSN, its antibacterial activity against *S. pyogenes* was tested using the Kirby–Bauer agar diffusion test. Concentrations of as-received NSN and its purified version were adjusted to the same level of active pharmaceutical ingredient for easier comparison (i.e., 25 µg/mL). Purified NSN was prepared using just the right amount of NSN powder (i.e., 25 µg for 1 mL of solution). However, in the case of as-received NSN, 40-times more substrate mass was necessary (i.e., 1000 µg for 1 mL of solution). The antibacterial efficacy of both samples was at the same level, confirming that the purification process did not affect the antimicrobial properties of NSN (see Figure 1). As a result, the purified NSN was used for the fabrication of NP_NSN.

Since NSN is a polypeptide, its processing into lipid NP should be performed at mild temperatures. Thus, the double emulsification/solvent evaporation method was selected for NP_NSN fabrication. NSN was introduced to lipid NP in the form of an aqueous inner phase (W_1_), which was emulsified with stearic acid dissolved in chloroform (O) to ensure uniform distribution of NSN within the lipid matrix as obtained primary emulsion W_1_/O was then emulsified in the external aqueous phase (W_2_) to form NP_NSN. To evaluate how the addition of NSN influenced the morphology and properties of NP, unloaded NP were also made. It was found out that the morphology of nanoparticles was not significantly influenced by NSN. However, NP_NSN were found smaller in diameter in comparison to NP. Nonetheless, both types of nanoparticles can be regarded as uniform in terms of their size, as the polydispersity index remained below 0.2 (see Figure 2a,b). Homogenous size distribution of the nanoparticles is necessary for their further uniform dispersion within the hydrogel matrix but also a more predictable and steady release of drug cargo [32]. A more pronounced difference was observed in terms of the zeta potential of the nanoparticles, since for unloaded NP it was strongly negative, while for NP_NSN is was close to −1 mV (see Figure 2c). This observation was the first indication of the successful loading of NSN into the nanoparticles since NSN is a cationic polypeptide. Similar observations were made by Chandrasekar et al. [33], who observed a higher zeta potential in NSN-loaded chitosan/alginate microparticles in comparison to unloaded microparticles.

Further investigation using an OPA-based amine detection method confirmed the high encapsulation efficacy of NSN (EE above 80%). Since this study was focused more on hydrogel composite wound dressing, no testing was performed to increase NSN loading in the nanoparticles. However, it has to be mentioned that further increase in NSN concentration within nanoparticles could be increased by modification of manufacturing procedure (e.g., increased initial concentration of NSN). Finally, the developed nanoparticles were tested in vitro in contact with L929 fibroblasts. It was found out that only the highest concentration of NP_NSN (i.e., 1000 µg/mL) was cytotoxic for the cells. However, around 65% of cells remained viable (see Figure 2d). Unloaded NP were cytocompatible in all tested concentrations. It has to be noted that 1000 µg/mL of NP is a relatively high dose for in vitro trials, and such a high ratio of nanoparticles to cells would be difficult to achieve in vivo.

Having successfully produced NP_NSN with uniform size distribution and sufficient NSN loading, further studies were carried out to fabricate GG and GG/Alg composite wound dressings. Both hydrogels can be prepared in the same conditions regarding the solvent solution (water), concentration, and the temperature necessary for proper dissolution of either GG or Alg, which ease the formation of the GG/Alg mixture [29]. For each hydrogel, two types of samples were prepared—one containing pure NSN and one containing NSN loaded into lipid NP (concentration of NSN was equal in both types of samples). Both GG and Alg can be cross-linked with divalent ions, such as Ca^2+^, so CaCl_2_ was used for hydrogel cross-linking [34,35]. Not only the procedure is non-toxic, but it was also proved that Alg cross-linking with Ca^2+^ contributes to hemostasis since Ca^2+^ ions can be released from the wound dressing and exchanged with Na^+^ ions present in the blood. Released Ca^2+^ ions induce platelet activation, play an important role in the coagulation cascade, and thus activate the coagulation process [36]. A similar phenomenon also occurs in GG [37].

The first evaluation of the composite wound dressings was performed just after sample preparation before the samples were freeze-dried. Rheological measurements of storage (G′) and loss (G″) moduli revealed significant differences between GG and GG/Alg. Both moduli were lower for GG/Alg, which indicates lower mechanical properties of that mixture compared to pure GG (see Figure 3a). The addition of NP_NSN did not influence G′ or G″ within GG and GG/Alg samples, although the concentration of the nanoparticles was high in comparison to the hydrogel itself. Nonetheless, all samples exhibited G′ > G″, indicating elastic solid behavior [35]. Upon drying, the samples were evaluated for their dry mass content to ensure complete incorporation of NP_NSN. For NSN-only loaded samples, the dry mass percentage was around 2% and for NP_NSN loaded materials it was above 3%, which corresponds nicely to hydrogel and NP_NSN content (2% *w*/*v* and 1% *w*/*v* in hydrated state, respectively) (see Figure 3b). CaCl_2_ contributed to only 0.1% w/v, even lower was the addition of free NSN (0.052% *w*/*v*). No significant variation in dry mass percentage was observed between samples taken from different parts of initially casted material. Thus, the uniform distribution of NP_NSN within the hydrogel matrix could be assumed.

The macroscopic appearance of all freeze-dried samples was similar—they remained intact during handling and could be easily cut into different sizes and shapes. After hydration in PBS, NSN-containing samples became translucent, while those loaded with NP_NSN were white and opaque, which can be attributed to the high concentration of lipid nanoparticles (see Figure 3c). SEM observations of sample cross-sections revealed huge discrepancies in the microstructure of all samples. Firstly, GG-based samples were highly porous. However, the pores were larger, irregular, and interconnected, while GG/Alg was characterized by a much finer microstructure with smaller and more uniform pores (see Figure 3d). As evidenced by Moreira et al. [38], GG microstructure can be refined by controlled freezing, i.e., alteration to freezing temperature or application of the insulated freezing device. Secondly, the surface of the pore wall in NSN-loaded samples was smooth. In the case of NP_NSN-containing samples, it was rough, which indicates that the nanoparticles or their agglomerates are entrapped within the material matrix.

Further investigation was devoted to the behavior of the developed materials in fluids since they are destined for application in infected areas, rich in exudate wounds. For that purpose, the materials were immersed in PBS and incubated at 37 °C to imitate physiological conditions. All materials showed a high capacity for fluid uptake as they were able to uptake 2000% more fluid as compared to their initial mass after freeze-drying (see Figure 4a). Slightly lower swelling was observed for NP_NSN-containing materials, but it can be attributed to the fact that those samples were characterized by a higher dry mass compared to the same sample volume for NSN-loaded materials. The same samples used for swelling tests were used for the determination of buffer pH (see Figure 4b). It was found out that in the presence of materials, buffer pH decreased over time. The lowest pH was observed for GG/Alg + NP_NSN, and that can be attributed to incomplete cross-linking of the material, its dissolution during incubation, and release of NP_NSN. This observation was confirmed by the measurements of the remaining mass after 48 h of incubation (see Figure 4c). It was found out that in the case of GG-based samples, mass loss was negligible (around 7% compared to initial mass). However, in the case of GG/Alg-based materials, sample mass decreased to around 70% and 60% of their initial mass for GG/Alg + NSN and GG/Alg + NP_NSN, respectively. Considering lower G′ observed for those samples in comparison to GG, it can be concluded that the concentration of CaCl_2_ was sufficient for complete gelation of GG, but it was not enough to fully cross-link Alg in GG/Alg mixture. That incomplete cross-linking leads to sample dissolution and release of unbound GG or Alg molecules, followed by the release of NP_NSN. The gradual degradation of those materials from the first few hours of incubation was also confirmed by a declining swelling ratio, suggesting a decline in sample mass.

The fastest and most effective NSN release was observed in GG + NSN due to the high porosity and large pore volume of the material (see Figure 4d). Encapsulation of NSN within lipid nanoparticles slowed down NSN release. However, after 24 h of incubation, almost the same amount of NSN was released as in the case of GG + NSN. Surprisingly, in the case of GG/Alg-based samples, NSN release was hampered, and only around 35% of NSN was released after 48 h of incubation. The release profile was not affected by NSN inclusion in lipid nanoparticles. This phenomenon can be related to the different microstructure of the hydrogel, as the diffusion of NSN by small and less interconnected pores might be difficult. Another explanation for this phenomenon might be that there is a chemical interaction between NSN and Alg that prevents NSN release. Zimet et al. [39] used Raman spectroscopy to prove that there was an interaction between COO^−^ groups in Alg and NH_3_^+^ groups present in NSN, indicating electrostatic interactions and ionic binding of NSN to Alg. Chandrasekar et al. [40] also observed that increased Alg concentration in alginate/chitosan films resulted in less effective NSN release. On the contrary, Guo et al. [41], who tested gellan gum/guar gum films loaded with NSN, did not detect any changes in infrared spectra between hydrogels and NSN. This suggests that no interaction occurred between NSN and functional groups present in GG so that NSN release from GG-based materials was more efficient.

Antibacterial activity of all developed materials was performed using exemplary Gram-positive *S. pyogenes*. GG and GG/Alg samples without NSN were not tested since it has already been proved that those polysaccharides do not have any antimicrobial activity themselves [29]. All materials showed significant inhibition of bacterial growth. However, some differences could be observed. Firstly, growth inhibition zones observed for GG-based materials were higher than in the corresponding GG/Alg samples (see Figure 5). That was contributed to a less effective NSN release from the latter ones. Secondly, in both types of hydrogels, bacterial growth inhibition was more effective in the case of NP_NSN-containing samples. We hypothesize that it was related to the fact that lipid nanoparticles provided thermal protection for NSN during sample preparation. Although the majority of NSN remained active (as evidenced by the formation of growth inhibition zones), the loss of activity could be easily avoided by the inclusion of NSN within lipid nanoparticles. Further studies will be performed to evaluate any structural changes in NSN induced by exposure to elevated temperatures. Our preliminary studies were focused on *S. pyogenes* only. However, other reports confirmed the antibacterial activity of NSN against *S. aureus* [42,43].

The cytotoxicity of the developed materials was evaluated in contact with L929 fibroblasts using metabolic activity assay, live/dead fluorescent staining, and wound healing assay (see Figure 6a–c). It was confirmed that 10% *w*/*v* extracts from the materials did not cause a significant decrease in cell viability and migration, except GG/Alg + NP_NSN. As it was evidenced before, those samples underwent fast degradation, presumably due to incomplete cross-linking of GG/Alg, and thus GG or Alg molecules together with NP_NSN could be released to extraction medium. As shown at the beginning (see Figure 2d), NP_NSN at a concentration above 500 µg/mL were cytotoxic for L929 cells. Thus, it was assumed that the extraction medium from GG/Alg + NP_NSN could contain a significant amount of NP_NSN that contributed to decreased cell viability. Intoxicated cells were also less likely to migrate. Therefore, wound healing was hampered in the case of GG/Alg + NP_NSN (see Figure 6c). The cytotoxicity of both GG and Alg was tested many times before and they were both regarded as non-toxic [44,45,46]. We believe that the observed toxic effect should be attributed to high concentrations of NP_NSN. However, it has to be confirmed by further, more detailed studies (e.g., involving evaluation of the number of nanoparticles released from GG/Alg + NP_NSN or their concentration in extraction medium).

Advantageously, as shown in cell adhesion assay (see Figure 6d), the cells were not adhering nor proliferating on the material surface, even though all conditions favouring cell adhesion were fulfilled, i.e., high density of seeded cells and sample immobilization preventing from floating or tilting in cell culture medium. This observation is of particular importance in terms of wound dressing materials as it is beneficial if the dressing does not adhere to the tissues, and a necessary dressing exchange procedure would not damage regenerating skin [10].

We believe that the developed materials are promising for wound healing application and the treatment of bacterial infections in wounds. In addition, the efficacy of the system can be easily tailored by encapsulation of other antimicrobials, such as more potent ABPs [47]. As ABPs have numerous advantages over traditional antibiotics, i.e., non-toxicity, slower emergence of bacterial resistance and broad antibacterial activity spectrum, they are commonly investigated in terms of antibacterial treatment [48]. Cathelicidin LL-37, the only human cathelicidin, is produced from precursor hCAP18 protein found in neutrophils. It has an α-helix structure, and due to its amphiphilic properties, it can interact with anionic bacterial membranes, leading to membrane leakage [49]. It was already proved that LL-37 has an antibacterial efficacy against both Gram-positive and Gram-negative bacteria, including *S. aureus*, *S. epidermidis* and *E. coli* [50,51]. As compared to NSN, LL-37 showed an increased rate of bacterial eradication, especially in the case of Gram-negative bacteria [50]. The antibacterial efficacy of ABPs can be further increased by the synthesis of novel peptides using natural ABPs as templates [52,53].

## 4. Materials and Methods

### 4.1. Nisin Purification

Nisin obtained from *Lactococcus lactis* (2.5% balance sodium chloride, Sigma-Aldrich, St. Louis, MO, USA) was dissolved in 30% acetonitrile with 0.1% TFA (Sigma-Aldrich, St. Louis, MO, USA). It was then filtered with a PTFE membrane (0.2 μm pores) to eliminate any kind of solid residues from the liquid. The purification was performed using Shimadzu preparative reverse-phase HPLC system with a Thermo Scientific Hypersil GOLD™ C18 column. The HPLC system was equipped with a UV-Vis detector which is set up to measure absorption at 215 nm and 280 nm wavelengths. The solutions used as mobile phases were: 30% acetonitrile, 0.1% TFA (phase A) and 98% acetonitrile, 0.1% TFA (phase B). During the purification, gradient elution was used, starting from 30% of phase B, increasing to 80% in 20 min. After that, from 20 to 25 min of elution, the concentration of phase B was maintained constant at 85%. Next, from 25 to 35 min of elution, the concentration was maintained constant at 30%. The fractions were collected in separate tubes and were further characterized using electrospray ionization mass spectrometry (ESI-MS). An amaZon ETD ion trap mass spectrometer (Bruker Daltonics, Bremen, Germany) with a standard electrospray (ESI) ion source was used. The optimal parameters were as follows: capillary voltage of −4 kV, the air was used as a nebulizing gas at 3 bar, the flow rate of drying gas set to 12 L/min, temperature of the heated capillary was adjusted to 300 °C; helium was used as collision gas.

Antimicrobial activity of NSN before and after purification process was assessed by Kirby–Bauer method (agar diffusion tests) using *Streptococcus pyogenes* (ATCC 12384, American Type Culture Collection, Manassas, VA, USA) as an example of Gram-positive bacteria. NSN solutions were prepared in sterile phosphate-buffered saline (PBS, Lonza, Basel, Switzerland) at a concentration of 1000 µg/mL for NSN as-received and 25 µg/mL for purified NSN (equal concentration of active NSN). The suspension of *S. pyogenes* was prepared in PBS at the concentration of 0.5 McFarland (1.5 × 10^8^ CFU/mL), and bacteria were seeded on Columbia agar with 5% sheep blood (Becton Dickinson). Two holes (5 mm in diameter) were cut in each plate, and 100 µL of NSN solution (either NSN as-received or NSN purified) was poured into the cavity. After 15 min at room temperature, the plates were moved to the incubator and kept at 37 °C for 24 h. After that, the diameter of inhibition of bacterial growth was measured. The experiment was run in a quadruplicate for each type of NSN solution.

### 4.2. Fabrication of Lipid Nanoparticles

NSN-loaded stearic acid nanoparticles were fabricated using the double emulsification/solvent evaporation method. In brief, 3.5 mg of purified NSN were dissolved in 100 µL of 2% *w*/*v* PVA (Mowiol 4–88, Mw = 31 kDa, Sigma-Aldrich, St. Louis, MO, USA) aqueous solution (W_1_). W1 was added dropwise to 2 mL of 2% *w*/*v* stearic acid (Sigma-Aldrich) solution in chloroform (O) (Avantor Performance Materials, Gliwice, Poland) and homogenized using an ultrasonic probe (VibraCell, Sonics, Newtown, CT, USA) for 90 s at 40% amplitude forming primary W_1_/O emulsion. Upon homogenization, W_1_/O was poured into 20 mL of ice-cold 2% *w*/*v* PVA aqueous solution (W2) and again homogenized using an ultrasound probe for 3 min at 40% amplitude. As prepared W_1_/O/W_2_ emulsion was mixed overnight using a magnetic stirrer at 500 rpm to allow chloroform evaporation. Obtained nanoparticles loaded with NSN (further referred to as NP_NSN) were collected and centrifuged at 20,000 rpm for 1 h at 5 °C (Sigma 3–30 K, Sigma, Osterode am Harz, Germany). The supernatant was preserved for determination of NSN encapsulation efficacy, while NP_NSN were resuspended in ultrapure water and centrifuged again. Rinsing with water was repeated 3 times. Purified NP_NSN were frozen at −80 °C for 24 h and then freeze-dried for 48 h. Freeze-dried NP were stored at −20 °C until further use.

Unloaded NP (further referred to as NP) were fabricated in the same way, while W_1_ contained only 100 µL of 2% *w*/*v* PVA aqueous solution without the addition of NSN.

### 4.3. Characterization of Lipid Nanoparticles

#### 4.3.1. Atomic Force Microscopy

Atomic force microscopy (AFM) was used for the characterization of NP morphology and shape. Freeze-dried NP were resuspended in ultrapure water (around 10 µg/mL), a drop of NP suspension was placed on a mica disc and air-dried at 37 °C for 24 h. Topographic images (scan size: 10 µm × 10 µm, resolution: 500 data points) were taken using an AFM microscope (Explorer, Thermomicroscopes) in contact mode using silicon nitride tip (MLCT-EXMT-A1, Vecco, spring constant k = 0.02 N/m). Gwydion 2.50 (Czech Metrology Institute, Brno, Czech Republic) software was used for image processing.

#### 4.3.2. Dynamic Light Scattering

NP size, polydispersity index, and surface zeta potential were evaluated by the dynamic light scattering (DLS) method using a ZetaSizer NanoZS (Malvern Instruments, Malvern, UK). Freeze-dried NP were resuspended in ultrapure water at around 1 mg/mL concentration and placed in polystyrene cuvettes (for size determination) or DTS1070 cuvettes (for zeta potential determination). Each measurement was performed in triplicate.

#### 4.3.3. NSN Encapsulation Efficacy

NSN encapsulation efficacy was determined using the supernatant after the first centrifugation of the nanoparticles. NSN concentration in the supernatant was measured using an o-phtalaldehyde (OPA) based amine detection method. The reaction mix was prepared using 6 mg of OPA (Sigma-Aldrich, St. Louis, MO, USA) added to 100 µL of methanol (Avantor Performance Materials, Gliwice, Poland) and 20 µL of 2-mercaptoethanol (Sigma-Aldrich, St. Louis, MO, USA). After complete dissolution of OPA, the mixture was poured into 10 mL of 400 mM boric acid solution (pH adjusted to 10.4 with the use of 5M NaOH, Avantor Performance Materials, Gliwice, Poland). An amount of 50 µL of the reaction mixture was mixed with 50 µL of supernatant sample in a black 96-well plate. After 10 min of incubation at room temperature and in the dark, fluorescence intensity was measured at λ_ex_ = 340 nm, λ_em_ = 450 nm. NSN encapsulation efficacy (EE) was calculated as follows:

$$\mathrm{Encapsulation}\;\mathrm{efficacy}\;\left(\%\right)=\frac{\mathrm{mass}\;\mathrm{of}\;\mathrm{NSN}\;\mathrm{in}\;\mathrm{nanoparticles}}{\mathrm{initial}\;\mathrm{mass}\;\mathrm{of}\;\mathrm{NSN}\;\mathrm{in}\;\mathrm{the}\;\mathrm{system}}\times 100\%,$$

While the following formula was used for the determination of drug loading (DL):

$$\mathrm{Drug}\;\mathrm{loading}\;\left(\%\right)=\frac{\mathrm{mass}\;\mathrm{of}\;\mathrm{NSN}\;\mathrm{in}\;\mathrm{nanoparticles}}{\mathrm{mass}\;\mathrm{of}\;\mathrm{nanoparticles}}\times 100\%.$$

EE and DL were determined in triplicate for three independent samples.

#### 4.3.4. Preliminary Evaluation of In Vitro Cytocompatibility

Preliminary evaluation of in vitro cytocompatibility of NP and NP_NSN was performed using L929 fibroblasts (ATCC^®^ CCL-1™, American Type Culture Collection, Manassas, VA, USA) according to ISO 10933-5 standard. The cells were cultured in Dulbecco’s modified Eagle’s medium (DMEM, PAN-Biotech, Aidenbach, Germany) supplemented with 10% fetal bovine serum (FBS, South America origin, PAN-Biotech, Aidenbach, Germany) and 1% penicillin/streptomycin (PAN-Biotech, Aidenbach, Germany). Cell culture was performed in a humidified atmosphere at 37 °C, with 5% CO_2_.

L929 cells were seeded in 100 µL cell culture medium at 10,000 cells/well in 96-well plate and allowed to adhere to the bottom of the plate. After 24 h, the cell culture medium was replaced with 100 µL of cell culture medium containing different concentrations of NP and NP_NSN ranging from 1 to 1000 µg/mL. Additionally, purified NSN was tested at the same concentrations. Cell viability was determined using resazurin-based metabolic activity assay (AlamarBlue reagent, 0.1 mg/mL dissolved in PBS, Sigma-Aldrich, St. Louis, MO, USA). After 24 h incubation in the presence of NSN, NP, or NP_NSN, the cell culture medium was withdrawn from wells, and 150 µL of cell culture medium containing 5% *v*/*v* AlamarBlue reagent was added into each well. After 3 h of incubation, 100 µL of medium from each well was transferred into a black 96-well plate. The fluorescence intensity was measured at λ_ex_ = 530 nm, λ_em_ = 590 nm using a microplate reader (FluoroSTAR Omega, BMG Labtech, Ortenberg, Germany). The percentage of resazurin reduction was calculated as follows:

$$\mathrm{Resazurin}\;\mathrm{reduction}\;\left(\%\right)=\frac{F_{x}-F_{0\%}}{F_{100\%}-F_{0\%}}\times 100\%,$$

where: F_x_ is the fluorescence of the sample, F_0%_ is the fluorescence of medium with AlamarBlue reagent without cells, F_100%_ is the fluorescence of completely reduced reagent (medium with the reagent autoclaved for 15 min at 121 °C). The experiment was performed in quadruplicate for each sample group. The cells cultured in a cell culture medium were used as a control sample.

### 4.4. Fabrication of Hydrogel Wound Dressings with NSN-Loaded NP

Gellan gum (GG, Sigma-Aldrich, St. Louis, MO, USA) and sodium alginate (Alg, Sigma-Aldrich, St. Louis, MO, USA) aqueous solutions were prepared by dissolving 2 g of GG or Alg in 80 mL of ultrapure water at 90 °C for 30 min in a water bath. GG solution was used as it was received. GG/Alg solution was prepared by combining equal volumes of GG and Alg solutions followed by vigorous mixing for 15 min using a horizontal shaker. Four mL of GG and GG/Alg solutions were transferred to new vials and placed again in a water bath at 65 °C, along with 1% *w*/*v* CaCl_2_ (Avantor Performance Materials, Gliwice, Poland) solution (cross-linking agent), the aqueous suspension of NP_NSN (10 mg/mL), the aqueous solution of NSN (0.52 mg/mL) and ultrapure water. Hydrogels containing NP_NSN were prepared as follows: 0.5 mL of NP_NSN suspension and 0.5 mL of CaCl_2_ solution were added into a vial containing GG or GG/Alg. The vial was vigorously mixed using a vortex mixer, cast into a polystyrene Petri dish (5 cm in diameter), and cooled down to 4 °C. For fabrication of hydrogels containing NSN alone, 0.5 mL of NP suspension was replaced by 0.5 mL of NSN solution, respectively. Final concentrations of all components were: 2% *w*/*v* GG or GG/Alg, 0.1% *w*/*v* CaCl_2_ (molar ratio between Ca^2+^ and -COOH groups set to around 1:5), 1% *w*/*v* NP + NSN (in GG + NP_NSN and GG/Alg + NP_NSN) or 0.052% *w*/*v* NSN (in GG + NSN and GG/Alg + NSN, NSN concentration was the same as NSN content in NP_NSN), corresponding to the hydrated state of the samples. The samples were then frozen at −80 °C for 24 h, freeze-dried for 48 h (Alpha 1-2, Martin Christ, Osterode am Harz, Germany), and stored at −20 °C until further use (except the samples used for rheological measurements—those were used immediately after preparation in the hydrated state).

### 4.5. Characterization of Hydrogel Wound Dressings with NSN-Loaded NP

#### 4.5.1. Rheological Properties

Rheological properties of unloaded hydrogels and hydrogels containing NP_NSN or NSN alone were determined using a rheometer (MCR, Anton Paar, Graz, Austria). For each sample group, 3 samples (20 mm in diameter, 1 mm in height) were tested. The measurements of storage modulus (G′) and loss modulus (G″) were performed in oscillatory mode at 0.1% strain sweeps and frequency of 1 Hz. The average G′ and G″ were calculated for all samples (*n* = 3).

#### 4.5.2. Gross Morphology

Freeze-dried samples were cut using a cylindrical cutter (12 mm in diameter) and visualized in a dry state using a digital microscope (Keyence VHX-7000, Keyence, Mechelen, Belgium) at 20× magnification. The samples were immersed in 3 mL of phosphate-buffered saline (PBS, VWR International) for 1 h and visualized again in a wet state.

#### 4.5.3. Scanning Electron Microscopy

The cross-sections of the samples were observed using scanning electron microscopy (SEM). Freeze-dried samples were cut into 1 mm slices and placed on aluminum holders using conductive carbon tape. Prior to SEM observations, the samples were sputter-coated with a 5 nm gold layer (EM ACE 600, Leica, Wetzlar, Germany). The samples were examined using a SEM microscope (Phenom XL, ThermoFisher Scientific, Waltham, MA, USA).

#### 4.5.4. Swelling, pH and, Mass Loss

Freeze-dried samples were cut into discs (12 mm in diameter, 2 mm in height), accurately weighed, immersed in 2 mL of PBS, and incubated at 37 °C for up to 48 h. At predetermined time points (i.e., 0.5 h, 1 h, 2 h, 4 h, 8 h, 24 h, and 48 h) the samples were withdrawn from PBS, excess PBS was gently removed from the surface using Kimtech wipes and weighed to determine their swelling capacity. Percentage swelling was calculated as follows:

$$\mathrm{Swelling}\;\left(\%\right)=\frac{M_{w}-M_{d}}{M_{d}}\times 100\%,$$

where: M_w_ is the mass of the wet sample, M_d_ is the mass of the dry sample. After that, the sample was transferred back into PBS.

In the meantime, 300 µL of the remaining PBS was transferred into a glass vial and its pH was measured using a pH meter (410, Elmetron, Zabrze, Poland) equipped with an electrode for low sample volume measurements (ERH-12-6, Elmetron, Zabrze, Poland). After the measurement, PBS was transferred into a sample vial and used for further incubation.

After 48 h of incubation and after the last weighting, the samples were extensively washed with ultrapure water (4 times, 5 mL of water, 3 min for each wash) and dried at 37 °C for 24 h. Dried samples were weighted, and percentage mass loss after 48 h of incubation was calculated as follows:

$$\mathrm{Remaining}\;\mathrm{mass}\;\left(\%\right)=\frac{M_{\deg}}{M_{\mathrm{non}-\deg}}\times 100\%,$$

where: M_deg_ is the mass of the dried sample after 48 h of incubation, M_non-deg_ is the initial mass of the sample (dry sample before incubation).

All experiments were performed in triplicate for each sample group.

#### 4.5.5. In Vitro NSN Release

The release of NSN was analyzed by the diffusion method. The freeze-dried samples were cut into cylinders (10 mm in height, 8 mm in diameter, estimated volume: 0.5 cm^3^) containing the same amount of NSN (i.e., 260 µg either added as free NSN in GG + NSN and GG/Alg + NSN or encapsulated in the nanoparticles in GG + NP_NSN and GG/Alg + NP_NSN). The samples were loaded into dialysis semipermeable membranes (MWCO 12 kDa, ZellutransRoth, Karlsruhe, Germany), sealed with clips from both sides, and immediately immersed in the vials containing 10 mL of PBS at pH = 7.4. The vials were then placed on a rotary shaker (20 rpm, VWR International, Radnor, PA, USA) at 37 °C. At predetermined time points (i.e., 0.5 h, 1 h, 2 h, 4 h, 8 h, 24 h, and 48 h) 200 µL of PBS was collected into an Eppendorf tube and kept at 4 °C until further use. The withdrawn volume of PBS in a sample vial was replaced with 200 µL of fresh PBS to retain constant buffer volume. The amount of NSN released was evaluated using an o-phtalaldehyde (OPA) based amine detection method in the same way as described before (Section 4.3.3). The experiment was performed in triplicate (3 samples for each sample group), 3 measurements were taken for each portion of the collected buffer.

#### 4.5.6. Antimicrobial Efficacy

The antimicrobial activity of the developed composite wound dressing was assessed by the Kirby–Bauer method (agar diffusion tests) using the same *S. pyogenes* strain as before. The suspension of *S. pyogenes* was prepared in PBS at the concentration of 0.5 McFarland (1.5 × 10^8^ CFU/mL), and bacteria were seeded on Columbia agar with 5% sheep blood (Becton Dickinson). Composite wound dressings were cut into discs (6 mm in diameter and 2 mm in height), sterilized via UV radiation for 25 min, and placed on agar. After 4 h at 4 °C (to allow sample hydration and diffusion of NSN), the plates were moved to the incubator and kept at 37 °C for 24 h. After that, the diameter of inhibition of bacterial growth was measured. The experiment was run in triplicate for each sample group. Antimicrobial susceptibility test disc (6 mm in diameter, Oxoid, Hampshire, UK) soaked with sterile PBS was used as a negative control.

#### 4.5.7. In Vitro Cytocompatibility

In vitro cytocompatibility of the samples was evaluated using L929 fibroblasts cultured as described before (Section 4.3.4). L929 cells were seeded in 100 µL cell culture medium at 10 000 cells/well in 96-well plate and allowed to adhere to the bottom of the plate. After 24 h, the cell culture medium was replaced with 100 µL of 10% *w*/*v* extracts from the samples in a cell culture medium (24 h extraction at 37 °C). Cell viability was determined using resazurin-based metabolic activity assay (as described in Section 4.3.4) and via live/dead fluorescence staining. For the live/dead staining, the cell culture medium was withdrawn from the wells and replaced with 100 µL of 0.1% calcein AM (Sigma-Aldrich) and 0.1% propidium iodide (Sigma-Aldrich) solution in PBS. After 20 min of incubation at 37 °C, the cells were visualized using a fluorescence microscope (Axiovert 40 CFL with HXP 120 C Metal Halide Illuminator, Zeiss, Jena, Germany) at 100× magnification.

A wound-healing assay was performed to evaluate the influence of sample extracts on cell migration and wound healing. L929 cells were seeded in 100 µL cell culture medium at 20,000 cells/well in a 96-well plate and allowed to adhere to the bottom of the plate. Once the cells reached almost complete confluence, a 200 µL pipette tip was used to create a wound at the bottom of the well (around 400 µm wide). The cells were washed twice with 200 µL of PBS to remove any detached cells. Phase-contrast images were taken for each well using an optical microscope at 100× magnification (Axiovert 40 CFL, Zeiss). PBS was then removed and replaced with 100 µL of sample extract. The cells were incubated in presence of the extracts for up to 3 days. Phase-contrast images were taken for each well on days 1, 2, and 3 of incubation. The images were analyzed using ImageJ software (National Institutes of Health) for the quantitative determination of the area of the wound at each time point.

A cell adhesion test was performed to assess whether the cells would adhere to the developed composite wound dressing. The freeze-dried samples were cut into discs (12 mm in diameter, 2 mm in height), placed in 24-well plates, and pressed with sterilized glass tubes (inner diameter of 10 mm) to prevent floating in the cell culture medium. Then, 20,000 L929 cells suspended in 1 mL of cell culture medium were poured into each well. After 24 h of incubation, the cell culture medium was withdrawn, and live/dead staining was performed in the same way as described before (1 mL of stain solution was used for each well).

### 4.6. Statistical Analyses

Statistical analyses of obtained data were performed using a one-way analysis of variance (one-way ANOVA) followed by Tuckey’s post hoc test. The assumptions of normal distribution and equal variance were verified using the Shapiro–Wilk and Levene median test, respectively (*p*-value > 0.05). The analyses were performed using OriginPro2020 software. The results were presented as mean ± standard deviation (SD).

## 5. Conclusions

Our study showed that spongy wound dressings based on polysaccharide hydrogel GG (i.e., GG + NSN and GG + NP_NSN) were able to uptake a significant volume of fluids, did not degrade over 48 h observation, and were able to release over 70% of its NSN cargo in a sustainable manner. Further, they were not cytotoxic for L929 fibroblasts, neither impaired cell migration nor wound healing. Prolonged-release of active NSN and thus increased antimicrobial efficacy were ensured by the inclusion of NSN-loaded lipid nanoparticles within GG matrix in GG + NP_NSN samples. We believe that the developed materials are promising for wound healing application and the treatment of bacterial infections in wounds. Their efficacy might be further improved, e.g., via encapsulation of more potent ABPs within lipid nanoparticles.

## Data Availability

Not applicable.
